# The interplay between host genetics and the gut microbiome reveals common and distinct microbiome features for complex human diseases

**DOI:** 10.1186/s40168-020-00923-9

**Published:** 2020-10-08

**Authors:** Fengzhe Xu, Yuanqing Fu, Ting-yu Sun, Zengliang Jiang, Zelei Miao, Menglei Shuai, Wanglong Gou, Chu-wen Ling, Jian Yang, Jun Wang, Yu-ming Chen, Ju-Sheng Zheng

**Affiliations:** 1 Zhejiang Provincial Laboratory of Life Sciences and Biomedicine, Key Laboratory of Growth Regulation and Translational Research of Zhejiang Province, School of Life Sciences, Westlake University, Hangzhou, China; 2 Guangdong Provincial Key Laboratory of Food, Nutrition and Health, Department of Epidemiology, School of Public Health, Sun Yat-sen University, Guangzhou, China; 3 Institute of Basic Medical Sciences, Westlake Institute for Advanced Study, Hangzhou, China; 4 Institute for Molecular Bioscience, The University of Queensland, QLD, Brisbane, Australia; 5 Institute for Advanced Research, Wenzhou Medical University, Wenzhou, 325027 Zhejiang China; 6 CAS Key Laboratory for Pathogenic Microbiology and Immunology, Institute of Microbiology, Chinese Academy of Sciences, Beijing, China; 7 MRC Epidemiology Unit, University of Cambridge, Cambridge, UK

**Keywords:** Gut microbiome, Host genetics, Bidirectional Mendelian randomization analyses, Disease-microbiome features

## Abstract

**Background:**

Interest in the interplay between host genetics and the gut microbiome in complex human diseases is increasing, with prior evidence mainly being derived from animal models. In addition, the shared and distinct microbiome features among complex human diseases remain largely unclear.

**Results:**

This analysis was based on a Chinese population with 1475 participants. We estimated the SNP-based heritability, which suggested that *Desulfovibrionaceae* and *Odoribacter* had significant heritability estimates (0.456 and 0.476, respectively). We performed a microbiome genome-wide association study to identify host genetic variants associated with the gut microbiome. We then conducted bidirectional Mendelian randomization analyses to examine the potential causal associations between the gut microbiome and complex human diseases. We found that *Saccharibacteria* could potentially decrease the concentration of serum creatinine and increase the estimated glomerular filtration rate. On the other hand, atrial fibrillation, chronic kidney disease and prostate cancer, as predicted by host genetics, had potential causal effects on the abundance of some specific gut microbiota. For example, atrial fibrillation increased the abundance of *Burkholderiales* and *Alcaligenaceae* and decreased the abundance of *Lachnobacterium*, *Bacteroides coprophilus*, *Barnesiellaceae*, an undefined genus in the family *Veillonellaceae* and *Mitsuokella*. Further disease-microbiome feature analysis suggested that systemic lupus erythematosus and chronic myeloid leukaemia shared common gut microbiome features.

**Conclusions:**

These results suggest that different complex human diseases share common and distinct gut microbiome features, which may help reshape our understanding of disease aetiology in humans.

Video Abstract

## Background

Ever increasing evidence has suggested that the gut microbiome is involved in many physiological processes, such as energy harvesting, the immune response and neurological function [1–3]. With successes of investigation into the clinical application of faecal transplants, the modulation of the gut microbiome has emerged as a potential treatment option for some complex diseases, including inflammatory bowel disease and colorectal cancer [4, 5]. However, it is still unclear whether the gut microbiome has the potential to be clinically applied for the prevention or treatment of many other complex diseases. Therefore, it is important to clarify the bidirectional causal association between the gut microbiome and complex human diseases or traits.

Mendelian randomization (MR) is a method that uses genetic variants as instrumental variables to investigate the causality between an exposure and an outcome in observational studies [6]. Prior studies provide evidence that the composition or structure of the gut microbiome can be influenced by host genetics [7–10]. On the other hand, host genetic variants associated with the gut microbiome are rarely explored in Asian populations; thus, we still lack instrumental variables to perform MR for the gut microbiome in Asians. This calls for a novel microbiome genome-wide association study (GWAS) in Asian populations.

Along with the causality issue between the gut microbiome and complex human diseases, it is unclear whether complex human diseases have similar or unique gut microbiome features. The identification of common and distinct gut microbiome features across different diseases may shed light on novel relationships among the complex diseases and update our understanding of the disease aetiology in humans. However, the composition and structure of the gut microbiome are influenced by a variety of factors, including the environment, diet and regional variation [11–13], which poses a key challenge for the description of representative microbiome features for a specific disease. Although there were several studies comparing disease-related gut microbiome features [14–16], few of them examined and compared the microbiome features across different complex human diseases.

In the present study, we performed a microbiome GWAS in a Chinese cohort, the Guangzhou Nutrition and Health Study (GNHS) [17], including 1475 participants. Subsequently, we applied a bidirectional MR method to explore the genetically predicted relationship between the gut microbiome and complex human diseases. To explore novel relationships among complex human diseases based on the gut microbiome, we investigated the shared and distinct gut microbiome features across diverse complex human diseases.

## Results

### Overview of the study

**Fig. 1:**
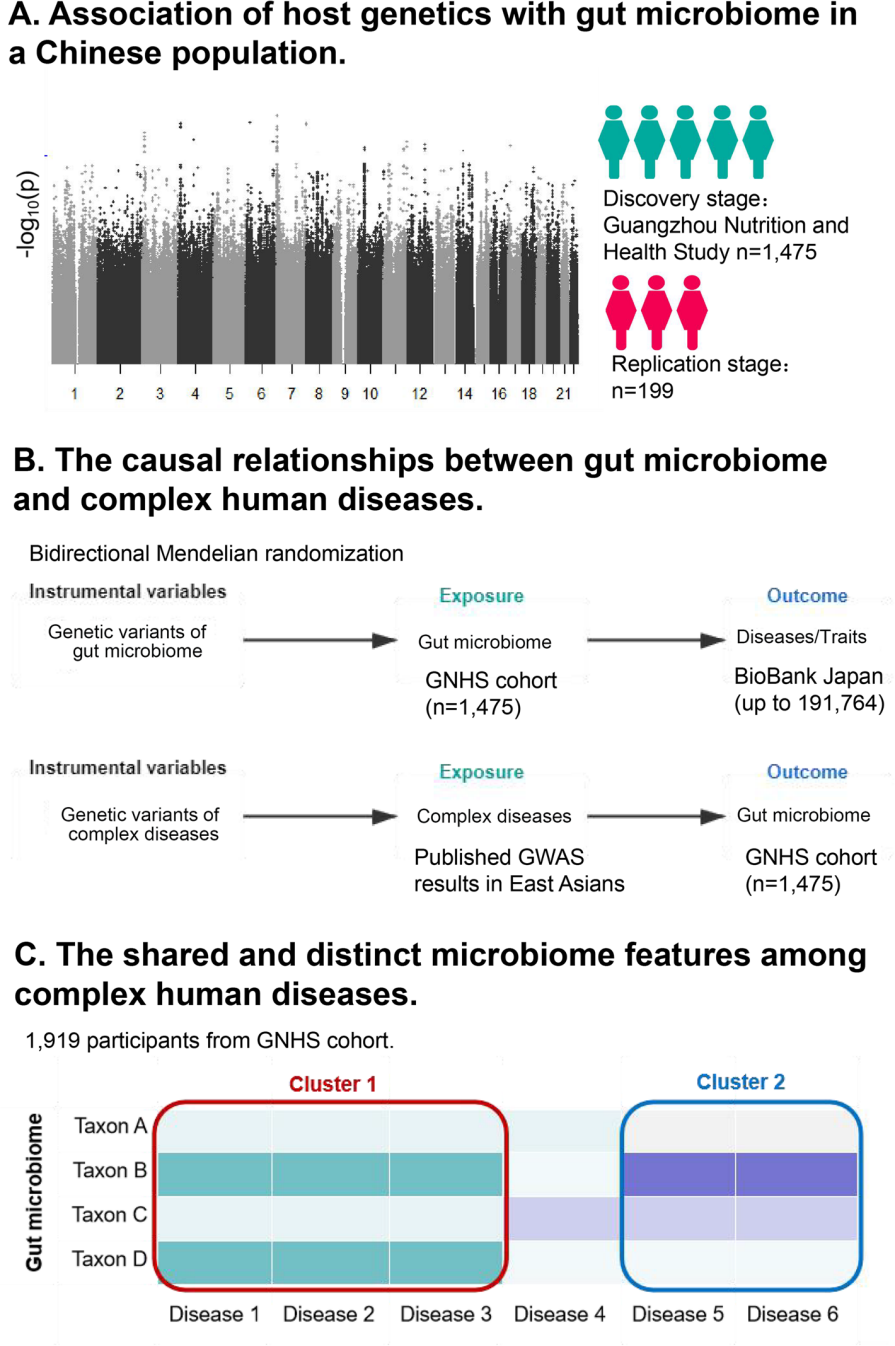
Study overview. The figure shows the highlights of our study. First, we performed a microbiome genome-wide association study in a Chinese population (step A). We validated significant genetic variants reported in previous studies and replicated our results in an independent cohort. Second, we investigated the causal relationship between the gut microbiome and complex human diseases using host genetics as instrumental variables for bidirectional Mendelian randomization (MR) analysis (step B). For the analysis of the effects of the gut microbiome on complex traits, we used publicly available GWAS summary statistics of complex traits (*n* = 58) and diseases (type 2 diabetes mellitus (T2DM), atrial fibrillation (AF), colorectal cancer (CRC) and rheumatoid arthritis) reported by BioBank Japan [19–24]. For the reverse MR analyses, the diseases of interest included T2DM (cases: 7,109; non-cases: 86,022), AF (cases: 8,180; non-cases: 28,612), coronary artery disease (cases: 1,515; non-cases: 5019), chronic kidney disease (*n* = 71,149), Alzheimer’s disease (cases: 477; non-cases: 442), CRC (cases: 8027; non-cases: 22,577) and prostatic cancer (cases: 495; non-cases: 640) reported in the previous large-scale GWASs in East Asians [19, 25–30]. Finally, we identified common and distinct gut microbiome features across different diseases (step C)

Our study was based on the GNHS, with 4048 participants (40–75 years old) living in the urban area of Guangzhou city recruited during 2008 and 2013 [17]. In the GNHS, stool samples were collected among 1937 participants during follow-up visits, among whom 1475 unrelated participants not taking antibiotics were included in our discovery microbiome GWAS. We then included an additional 199 participants with both genetic data and gut microbiome data as a replication cohort, which belonged to the control arm of a case-control study of hip fracture in Guangdong Province, China [18] (see also Fig. 1).

### SNP-based heritability of the gut microbiome

**Fig. 2:**
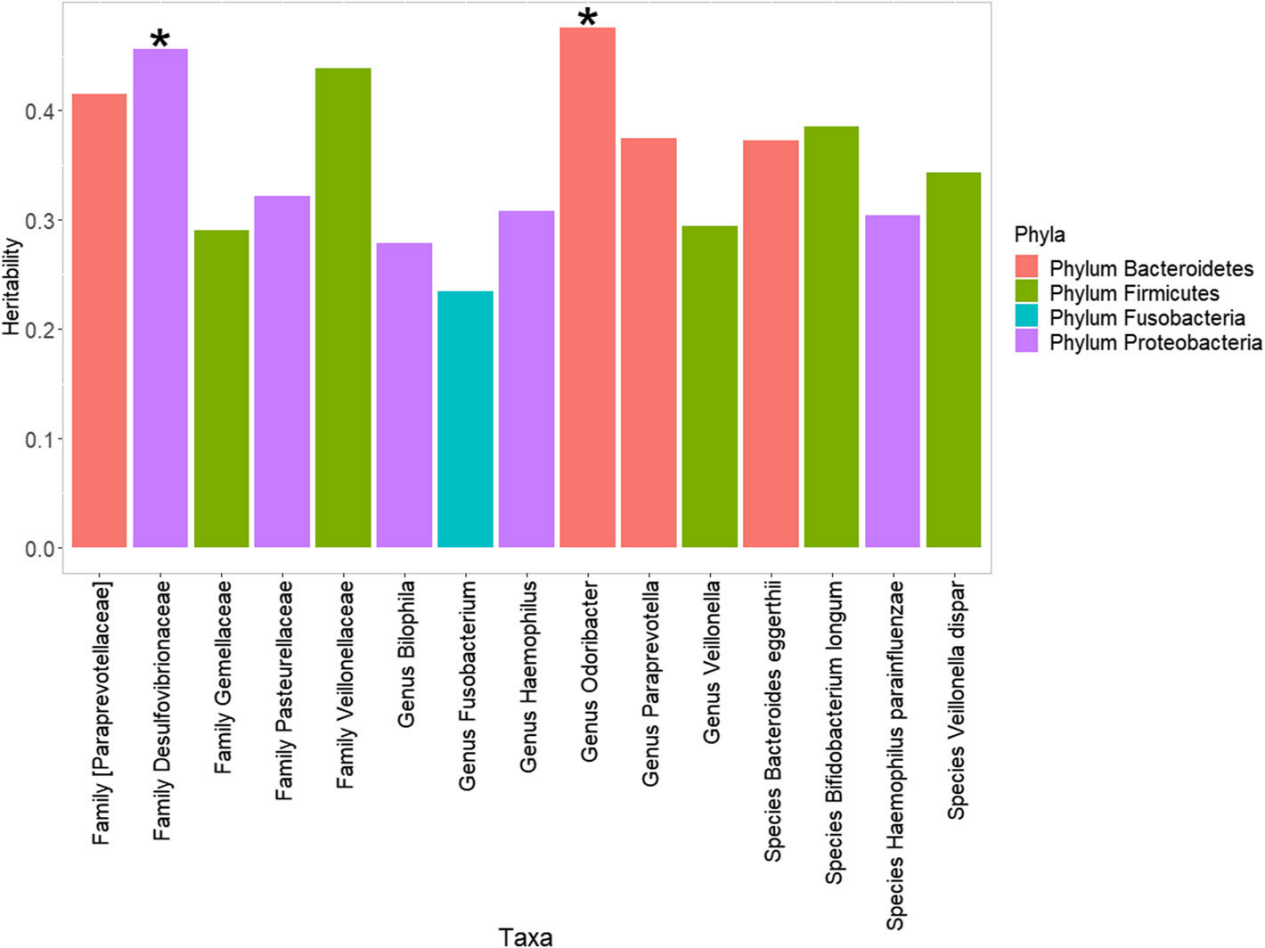
The SNP-based heritability of the gut microbiome. The plot shows the taxa with nominally significant heritability estimates (*p* < 0.05). *p < 0.05/*n*, where *n* is the effective number of independent taxa in each taxonomic level

The heritability of alpha diversity ranged from 0.035 to 0.103 (SE: from 0.174 to 0.193, Supplementary Table S3). Significant heritability estimates were observed for several taxa (see also Fig. 2, Supplementary Table S3), with crude *p* values *<* 0.05. To further correct the multiple testing, we calculated the effective number of independent taxa in each taxonomic level (phylum level: 2.3, class level: 2.9, order level: 2.9, family level: 5.5, genus level: 5.6, species level: 3.2), as some taxa were highly correlated with each other. The results suggested that *Desulfovibrionaceae* and *Odoribacter* were heritable (*p <* 0.05/*n*, where *n* is the effective number of independent taxa). Notably, among the suggestively heritable taxa in our cohort [*Paraprevotellaceae*], *Veillonellaceae*, *Desulfovibrionaceae*, *Pasteurellaceae*, *Odoribacter*, *Paraprevotella*, *Veillonella* and *Bifidobacterium* had nominally significant heritability estimates in prior literature [7, 31–33].

### Association of host genetics with gut microbiome features

We generated categorical variable enterotypes (*Prevotella* vs. *Bacteroides*) of the participants based on the genus-level relative abundance of the gut microbiome [34]. Thereafter, we performed a GWAS for enterotypes using a logistic regression model to explore potential associations between host genetics and enterotypes. However, we did not find any genome-wide significant loci (*p <* 5 × 10^−8^).

To examine the association of host genetic variants with alpha diversity, we performed a GWAS for four indices (Shannon diversity index, Chao1 diversity indices, observed OTU index and phylogenetic diversity), but again, no genome-wide significant signal (*p <* 5 × 10^−8^) was found. To further investigate whether there is a host genetic basis underlying alpha diversity, we constructed a polygenic score for each alpha diversity indicator in the replication cohort using the genetic variants that showed suggestive significance (*p <* 5 × 10^−5^) in the discovery GWAS. The polygenic score was not significantly associated with its corresponding alpha diversity index in our replication cohort. Furthermore, none of the associations with alpha diversity indices reported in the literature could be replicated (Supplementary Table S8) [7].

The beta diversity GWAS was performed with MicrobiomeGWAS based on Bray–Curtis dissimilarity [35]. We found that one locus at the *SMARCA2* gene (rs6475456) was associated with beta diversity at a genome-wide significant level (*p =* 3.96 × 10^−9^). However, we could not replicate the results in the replication cohort, which might be due to the limited sample size of the replication cohort. In addition, prior literature had reported 73 genetic variants that were associated with beta diversity [8, 13, 36, 37], among which we found that 3 single-nucleotide polymorphisms (SNPs, *UHRF2* gene-rs563779, *LHFPL3* gene-rs12705241, *CTD-2135J3*.*4*-rs11986935) had nominally significant (*p <* 0.05) associations with beta diversity in our cohort (Supplementary Table S7), although none of the associations survived Bonferroni correction. These studies used various methods for the sequencing and calculation of beta diversity, which raised challenges to verify and extrapolate results across populations.

We subsequently performed a discovery GWAS for individual gut microbes in our own GNHS discovery dataset. For the taxa (*n* = 114) present in not fewer than 90% of participants, we carried out an analysis based on a log-normal model. For other taxa (*n* = 88) present in fewer than 90%, we transformed the absence/presence of the taxon into binary variables and used a logistic model to prevent zero inflation (Supplementary Table S1). For all the gut microbiome taxa, the significance threshold was defined as 5 × 10^−8^ in the discovery stage. We found that 6 taxa were associated with host genetic variants in the discovery cohort (*p <* 5 × 10^−8^/*n*, where *n* is the effective number of independent taxa in each taxonomic level, Supplementary Table S5); however, these associations were not significant (*p* > 0.05) in the replication cohort. We then took the genetic loci reported to be associated with individual taxa in prior studies [7, 8, 13, 37] for replication in our GNHS dataset. Although none of the associations of these genetic variants with taxa survived the Bonferroni correction (*p <* 1 × 10^−4^), we found that *STPG2*-rs4699323 had a nominally significant association (*p <* 0.05) with *Clostridiales* (Beta: − 0.131 [95% CI − 0.233, − 0.029], *p =* 0.012; Supplementary Table S6). We then used a threshold of *p <* 5 × 10^−5^ at the discovery GWAS stage to incorporate additional genetic variants that might explain a larger proportion of heritability for taxa, and based on this, we constructed a polygenic score for each taxon in the replication. We found that the polygenic scores were significantly associated with 5 taxa, including *Saccharibacteria* (also known as *TM7* phylum), *Clostridiaceae*, *Comamonadaceae*, *Klebsiella* and *Desulfovibrio d168*, in the replication set (*p <* 0.05, Methods, see also Supplementary Figure S1, Supplementary Table S9).

### Genetic correlation of gut microbiome and traits

While the associations of the microbiome with complex diseases and traits have been widely reported [38], the genetic correlation between the gut microbiome and traits of interest is less clear. Therefore, we applied bivariate GREML analysis to address this question. The traits included BMI, fasting blood sugar (FBS), glycosylated haemoglobin (HbA1c), systolic blood pressure (SBP), diastolic blood pressure (DBP), high-density lipoprotein cholesterol (HDL-C), low-density lipoprotein cholesterol (LDL-C), total cholesterol (TC) and triglyceride (TG), none of which could pass Bonferroni correction. HDL-C was the only trait that had nominal genetic correlation (*p <* 0.05) with gut microbes (specifically, *Desulfovibrionaceae* and [*Prevotella*], Supplementary Table S4).

### Bidirectional assessment of the genetically predicted association between the gut microbiome and complex diseases/traits

Using genetic-variant-composed polygenic scores as genetic instruments, we performed MR analysis to assess the putative causal effect of the microbiome (*Saccharibacteria*, *Clostridiaceae*, *Comamonadaceae*, *Klebsiella* and *Desulfovibrio d168*) on complex human diseases or traits. The inverse variance weighted (IVW) method was used for the MR analysis, while the other three methods (weighted median, MR-Egger and MR-PRESSO) were applied to confirm the robustness of the results. Horizontal pleiotropy was assessed using the MR-PRESSO global test and MR-Egger regression. For the analysis of the gut microbiome on complex traits, we downloaded publicly available GWAS summary statistics of complex traits (*n* = 58) and diseases (type 2 diabetes mellitus (T2DM), atrial fibrillation (AF), colorectal cancer (CRC) and rheumatoid arthritis (RA)) reported by BioBank Japan [19–24]. The results suggested that *Saccharibacteria* (per 1-SD higher in the log-transformed abundance) could potentially decrease the concentration of serum creatinine (− 0.011 [95% CI − 0.019, − 0.003], *p =* 0.007) and increase the estimated glomerular filtration rate (eGFR) (0.012 [95% CI 0.004, 0.020], *p =* 0.003, Supplementary Table S10), which might help improve renal function. We did not find evidence of pleiotropic effects: genetic variants associated with *Saccharibacteria* were not associated with any of the above traits (58 complex traits and 4 disease outcomes, *p <* 0.05/62). These taxa were not causally associated with other complex diseases or traits in our MR analyses, which might be due to the limited genetic instruments discovered in our present study.

**Fig. 3:**
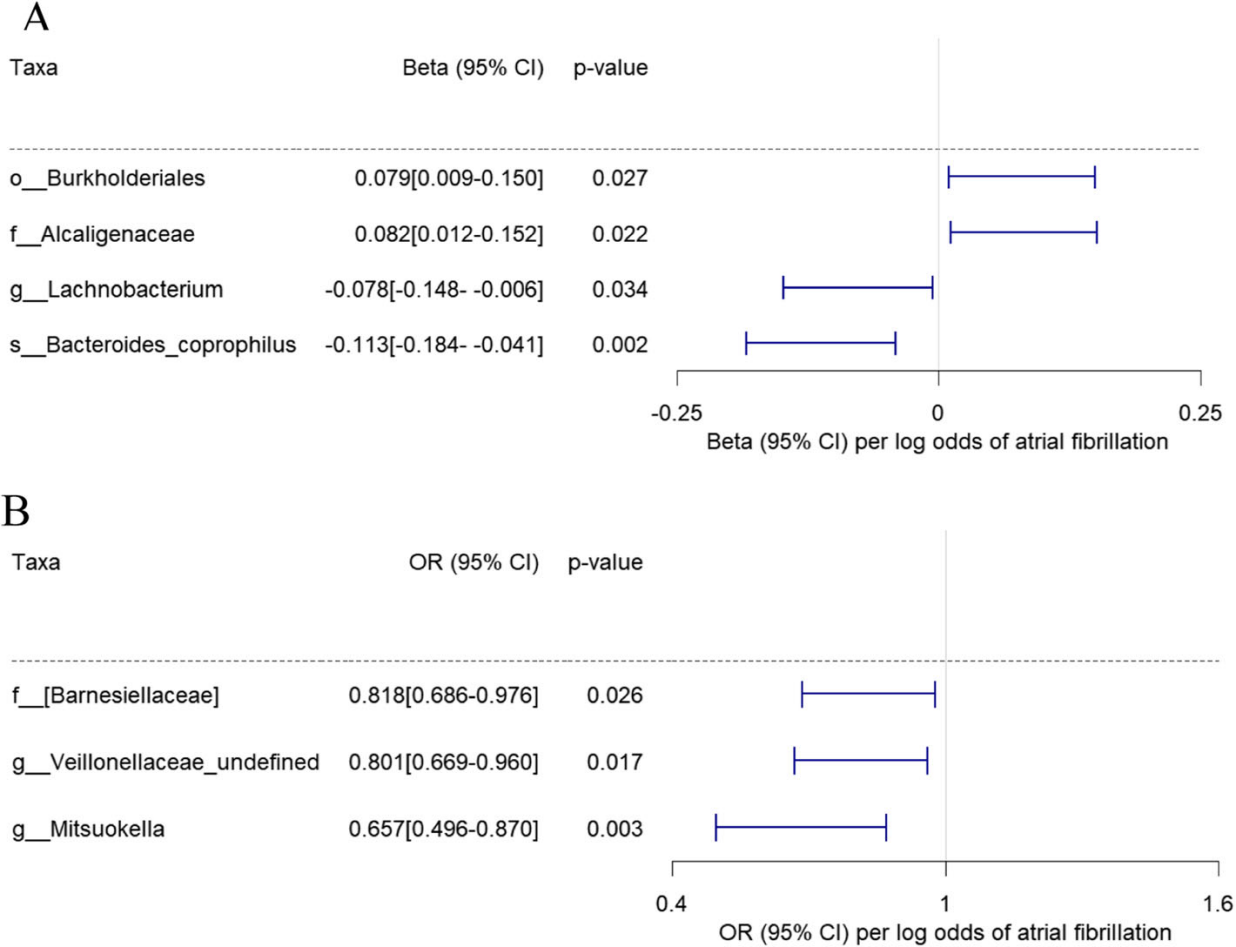
Effect of host genetically predicted higher atrial fibrillation risk on the gut microbiome. **a** Causal association of atrial fibrillation with the abundance of *Burkholderiales*, *Alcaligenaceae*, *Lachnobacterium* and *Bacteroides coprophilus*. The magnitude of the effect of atrial fibrillation on taxa is dependent on changes in the abundance of bacteria (1-SD of the log-transformed abundance) per genetically determined higher log odds of atrial fibrillation. **b** Causal association of atrial fibrillation with the presence of *Barnesiellaceae*, an undefined genus in the family *Veillonellaceae* and *Mitsuokella*. The magnitude of the effect of atrial fibrillation on taxa is presented as an odds ratio increase in the log odds of atrial fibrillation

We subsequently performed a reverse MR analysis to assess the potential causal effect of complex human diseases on gut microbiome features. For the reverse MR analyses, the diseases of interest included T2DM, AF, coronary artery disease (CAD), chronic kidney disease (CKD), Alzheimer’s disease (AD), CRC and prostatic cancer (PCa), and their instrumental variables for the MR analysis were based on previous large-scale GWASs in East Asians [19, 25–30]. The results suggested that AF and CKD were causally associated with the gut microbiome (see also Fig. 3a, b, Supplementary Table S11). Specifically, genetically predicted higher risk of AF (per log odds) was associated with a lower abundance of *Lachnobacterium* (Beta: − 0.078 [95% CI − 0.148, − 0.006], *p =* 0.034), *Bacteroides coprophilus* (Beta: − 0.113 [95% CI − 0.184, − 0.041], *p =* 0.002), *Barnesiellaceae* (odds ratio: 0.818 [95% CI 0.686, 0.976], *p =* 0.026), an undefined genus in the family *Veillonellaceae* (odds ratio: 0.801 [95% CI 0.669, 0.960], *p =* 0.017) and *Mitsuokella* (odds ratio: 0.657 [95% CI 0.496, 0.870], *p =* 0.003), and higher abundance of *Burkholderiales* (Beta: 0.079 [95% CI 0.009, 0.150], *p =* 0.027) and *Alcaligenaceae* (Beta: 0.082 [95% CI 0.012, 0.152], *p =* 0.022). Additionally, genetically predicted higher risk of CKD could increase *Anaerostipes* (Beta: 0.291 [95% CI 0.057, 0.524], *p =* 0.015) abundance, and a higher risk of PCa could decrease [*Prevotella*] (odds ratio: − 0.758 [95% CI − 1.354, − 0.162], *p =* 0.013).

### Microbiome features of complex human diseases

To further investigate the potential complex diseases that may be correlated with the taxa affected by AF, we applied Phylogenetic Investigation of Communities by Reconstruction of Unobserved States (PICRUSt) to predict the disease pathway abundance [39]. We used Spearman’s rank-order correlation to test whether the relative abundances of predicted diseases based on PICRUSt were associated with the aforementioned AF-associated taxa (see also Supplementary Figure S2, Supplementary Table S12). The heatmap indicated that cancers and neurodegenerative diseases, including Parkinson’s disease (PD), AD, amyotrophic lateral sclerosis (ALS) and AF, were correlated with similar gut microbiomes. Although the association among these diseases is highly supported by previous studies [40–42], no study has compared common gut microbiome features across these different diseases.

**Fig. 4:**
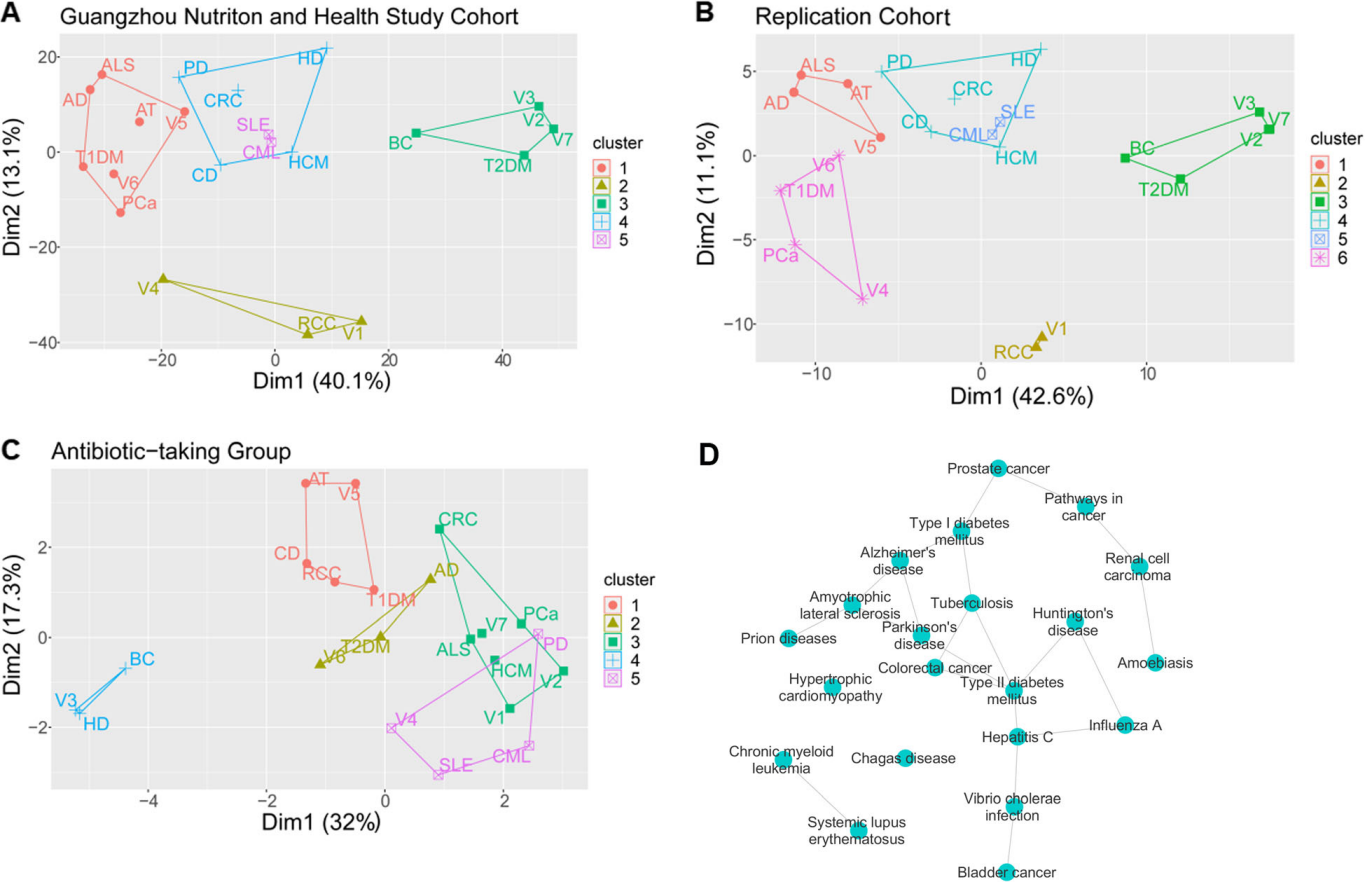
Association and cluster of diseases predicted by the gut microbiome. **a** Plot of clusters in the Guangzhou Nutrition and Health Study (GNHS) cohort (*n* = 1919). **b** Plot of cluster results in the replication cohort (*n* = 217). **c** Plot of 5 clusters in antibiotic-taking participants (*n* = 18). The optimal cluster was 5 in the GNHS cohort and 6 in the replication cohort. The clusters share consistent components between the two studies. In contrast, components are different between antibiotic-taking participants and control groups. Dimension1 (Dim1) and dimension2 (Dim2) explained 40.1% and 13.1% of the variance, respectively, in the GNHS cohort. The annotation for variables is as follows. *AT* African trypanosomiasis, *AD* Alzheimer’s disease, *V1* amoebiasis, *ALS* amyotrophic lateral sclerosis, *BC* bladder cancer, *CD* Chagas disease, *CML* chronic myeloid leukaemia, *CRC* colorectal cancer, *V2* hepatitis C, *HD* Huntington’s disease, *HCM* hypertrophic cardiomyopathy, *V3* influenza A, *PD* Parkinson’s disease, *V4* pathways in cancer, *V5* Prion disease, *PCa* prostate cancer, *RCC* renal cell carcinoma, *SLE* systemic lupus erythematosus, *V6* tuberculosis, *T1DM* type I diabetes mellitus, *T2DM* type II diabetes mellitus, *V7 Vibrio cholerae* infection. **d**. Gut microbiome-predicted network of relationships among different complex human diseases. The relationship between diseases is determined by SPIEC-EASI with non-normalized predicted abundance data. The diseases that shared the same edge had the gut microbiome-predicted correlation

To compare gut microbiome features across human diseases, we used the predicted disease abundance based on PICRUSt and performed k-medoid clustering. According to the optimum average silhouette width [43], we chose the optimal number of clusters for further analysis. The plot showed that neurological diseases, including ALS and AD, belonged to the same cluster, while PD and CRC had much similarity in the gut microbiome. The results also suggested that systemic lupus erythematosus (SLE) and chronic myeloid leukaemia (CML) shared similar gut microbiome features (see also Fig. 4a, b). Moreover, we could replicate these clusters in our replication cohort, which suggested that the clustering results were robust (see also Fig. 4c).

**Fig. 5:**
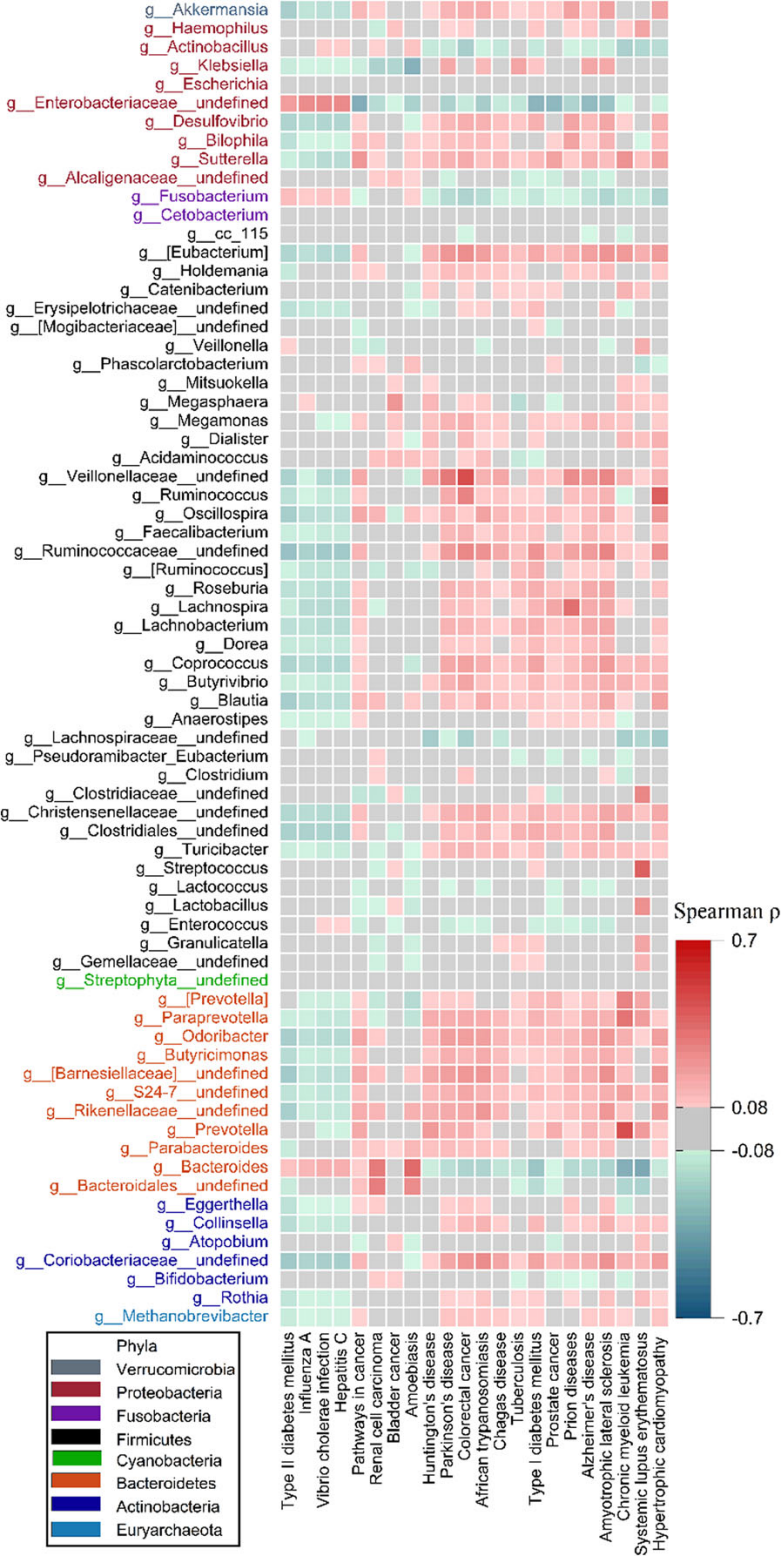
Correlation of complex human diseases with the gut microbiome. The heatmap shows Spearman’s correlation of predicted diseases and the gut microbiome at the genus level. The grey components show no significant correlation with Bonferroni correction (*p* > 0.05/(5.6*22), *p* > 0.0004)

We further asked whether the gut microbiome contributed to the novel clustering. To this end, we repeated the analysis among participants who took antibiotics less than two weeks before stool sample collection, considering that antibiotic treatments were believed to cause microbiome imbalance. We used the Jaccard similarity coefficient to estimate the cluster difference among the GNHS cohort, the replication cohort and the antibiotic group. The similarity between the GNHS cohort and the replication cohort was higher than that between the GNHS cohort and the antibiotic group (Jaccard similarity coefficient: 0.61 vs. 0.11). The results indicated a different clustering, which suggested that the gut microbiome indeed contributed to the correlations among diseases (see also Fig. 4d). To further demonstrate common microbiome features across different diseases, we examined the correlation of the predicted diseases with genus-level taxa. The results showed that complex human diseases shared similar gut microbiome features, as well as distinct features on their own (see also Fig. 5, Supplementary Table S13).

To validate whether the disease-related gut microbiome features annotated by KEGG would be associated with the risk of the disease in a real-world community-based cohort, we used T2DM as an example, examining the association of predicted T2DM-related microbiome features with T2DM risk in our GNHS cohort. We constructed a microbiome risk score (MRS) based on 16 selected taxa with predicted correlation coefficients with T2DM greater than 0.2. A logistic regression model was used to examine the association between MRS and T2DM risk in GNHS (*n* = 1886, with 217 T2DM cases). The results showed that MRS was positively associated with the risk of T2DM (odds ratio: 1.176 [95% CI 1.114, 1.244], *p =* 8.75 × 10^−9^).

## Discussion

Our study is among the first to investigate host genetics-gut microbiome associations in East Asian populations and reveals that several microbiome species (e.g., *Saccharibacteria* and *Klebsiella*) are influenced by host genetics. We found that *Saccharibacteria* might causally improve renal function by affecting renal function biomarkers (i.e., creatinine and eGFR). On the other hand, complex diseases such as atrial fibrillation, chronic kidney disease and prostate cancer have potential causal effects on the gut microbiome. More interestingly, our results indicated that different complex diseases may be mechanistically correlated by sharing common gut microbiome features but also maintaining their own distinct microbiome features.

Previous studies and our study showed that the gut microbiome had an inclination to be influenced by host genetics [8, 10, 37, 44, 45]. The results suggested that *Desulfovibrionaceae* and *Odoribacter* had nominally significant heritability estimates, which were consistent with prior results [7]. We also identified several suggestively heritable taxa that were nominally significant in previous studies [31–33]. In addition, we successfully constructed polygenic scores for *Clostridiaceae* and *Comamonadaceae*, both of which have been identified to be heritable or suggested to be heritable [7, 45].

We could not replicate any of the reported genetic variants that were significantly associated with gut microbiome features in prior reports, which might be due to multiple reasons. One of the major reasons may be that the massive multiple testing in insufficiently large samples in prior microbiome GWASs may potentially lead to false-positive findings. In addition, other factors, including ethnic differences, heterogeneity between studies, gene-environment interactions and dissimilarity in sequencing methods, might also make it difficult to extrapolate results from microbiome GWASs across populations in the microbiome field. Nevertheless, we successfully replicated several polygenic scores of the gut microbiome. The current study represents the largest dataset, to the best of our knowledge, in Asian populations and may serve as a unique resource for large-scale trans-ethnic meta-analyses of microbiome GWASs in the future.

MR analysis showed that *Saccharibacteria* might decrease the concentration of serum creatinine and increase eGFR. Little is known about *Saccharibacteria* as one of the uncultivated phyla, and previous studies have shown that it might be essential for the immune response, oral inflammation and inflammatory bowel disease [46–48]. Our results also provided a genetic instrument of *Saccharibacteria* for further causal analysis with other complex diseases. The reverse MR analysis provided evidence that AF, CKD and PCa could causally influence the gut microbiome. The rare and low-frequency variants may have an important impact on common diseases [49]; thus, it will be of interest to clarify the effects of low-frequency variants on the gut microbiome in cohorts with large sample sizes in the future.

Our results indicate that the gut microbiome helps reveal novel and interesting relationships among complex human diseases, and different diseases may have common and distinct gut microbiome features. A prior study including participants from different countries identified three microbiome clusters [34]. Notably, this study focused on classifying the individuals into distinct enterotypes regardless of the individuals’ health status, while in the present study, we described representative microbiome features for diseases of interest. We provided an approach to interpret the data from mechanistic studies based on the microbiome. The microbiome features revealed a close association of AF with neurodegenerative diseases as well as cancers, which was supported by prior studies showing that AF had a correlation with AD and PD [40, 41], and AF patients had relatively higher risks of several cancers, including lung cancer and CRC [42, 50]. We also observed that the microbiome features of SLE and CML were highly similar. Interestingly, a tyrosine kinase inhibitor of platelet-derived growth factor receptor, imatinib, was widely used to treat CML and significantly ameliorated survival in murine models of SLE [51]. In addition, a close association between CRC and PD has also been reported in several observational cohorts [52, 53]. Collectively, these findings strongly supported our hypothesis that complex human diseases sharing similar microbiome features might be mechanistically correlated. Furthermore, from the perspectives of risk genes of AF and neurodegenerative diseases, previous GWASs for AF identified two loci at *PITX2* gene*-*rs6843082 and *C9orf3* gene*-*rs7026071, which were also associated with a risk of ALS (*p =* 0.0138 and *p =* 0.049, respectively) [54–56].

We acknowledge several limitations of our study. First, the participants were of East Asian ancestry; thus, factors such as ethnic differences and gene-environment interactions might make it difficult to generalize the prior results to our study and extrapolate our results to different ethnic populations. Second, although our analysis included participants with the identical by descent (IBD) < 0.185, the vertical transmission of the microbiome from parent to offspring might still partially affect the SNP-based heritability estimates and polygenic scores [32, 57]. Third, genetic factors could explain only a small proportion of the variance in gut microbiome features; thus, the power to detect the causal relationship was limited. Therefore, large-scale studies are warranted to reveal potential relationships between the gut microbiome and complex traits.

## Conclusions

In summary, we reveal some causal relationships between the gut microbiome and complex human diseases or traits. The disease and gut microbiome feature analysis revealed novel relationships among complex human diseases, which may help reshape our understanding of disease aetiology and provide some clues for extending the clinical indications of existing drugs for different diseases.

## Method

### Study participants and sample collection

Our study was based on the Guangzhou Nutrition and Health Study (GNHS), with 4048 participants (40–75 years old) living in the urban area of Guangzhou city recruited during 2008 and 2013 [17]. We followed up with participants every 3 years. In the GNHS, stool samples were collected from 1937 participants during follow-up visits. Among those with stool samples, 1717 participants had genetic data, and IBD for 1475 participants was less than 0.185.

We included 199 participants with both genetic data and gut microbiome data as a replication cohort, which belonged to the control arm of a case-control study of hip fracture with the participants (52–83 years old) recruited between June 2009 and August 2015 in Guangdong Province, China [18].

Blood samples of all participants were collected after overnight fasting, and the buffy coat was separated from whole blood and stored at − 80°C. Stool samples were collected during the on-site visit of the participants at Sun Yat-sen University. All samples were manually stirred, separated into tubes and stored at − 80 °C within 4 h.

### Genotyping data

For both the discovery and replication cohorts, DNA was extracted from leukocytes using the TIANamp® Blood DNA Kit (DP348, TianGen Biotech Co, Ltd., China) according to the manufacturer’s instructions. DNA concentrations were determined using the Qubit quantification system (Thermo Scientific, Wilmington, DE, USA). Extracted DNA was stored at − 80 °C. Genotyping was carried out with Illumina ASA-750K arrays. Quality control and relatedness filters were performed by PLINK1.9 [58]. Individuals with a high or low proportion of heterozygous genotypes (outliers defined as 3 standard deviations) were excluded [59]. Individuals who had different ancestries (the first two principal components ± 5 standard deviations from the mean) or related individuals (IBD > 0.185) were excluded [59]. Variants were mapped to the 1000 Genomes Phase 3 v5 by SHAPEIT [60, 61], and then we conducted genome-wide genotype imputation with the 1000 Genomes Phase 3 v5 reference panel by Minimac3 [62, 63]. Genetic variants with imputation accuracy RSQR > 0.3 and MAF > 0.05 were included in our analysis. We used the Pan-Asian reference panel, consisting of 502 participants, and SNP2HLA v1.0.3 to impute the HLA region [64–66].

### Sequencing and processing of 16S rRNA gene data

Microbial DNA was extracted from faecal samples using the QIAamp® DNA Stool Mini Kit per the manufacturer’s instructions. DNA concentrations were determined using the Qubit quantification system. The V3–V4 region of the 16S rRNA gene was amplified from genomic DNA using primers 341F (CCTACGGGNGGCWGCAG) and 805R (GACTACHVGGGTATCTAATCC). At the step of amplicon generation, 2 μL sterile water was used as negative controls in the PCR reaction system. At the subsequent step of sequencing, no sequencing negative controls were included, since no contamination of PCR products was observed. The pooled amplicons were sequenced using MiSeq Reagent Kits v2 on the Illumina MiSeq System with 2 × 250 bp pair-end sequencing.

Fastq files were demultiplexed by MiSeq Controller Software. Ultra-fast sequence analysis (USEARCH) was performed to trim the sequence for amplification primers, diversity spacers, sequencing adapters and merged paired-end reads [67]. The low-quality reads (Phred quality scores ≤ 30) were removed. Operational taxonomic units (OTUs) were clustered based on 97% similarity using UPARSE [68]. We removed the OTUs present only in one sample. OTUs were annotated with Greengenes 13_8 (https://greengenes.secondgenome.com/) [69]. After randomly selecting 10000 reads for each sample, Quantitative Insights into Microbial Ecology (QIIME) software version 1.9.0 was used to calculate alpha diversity (Shannon diversity index, Chao1 diversity indices and the observed OTU index and phylogenetic diversity) based on the rarefied OTU counts [70].

### Statistical analysis

#### Proportion of variance explained by all SNPs

We used the GREML method in GCTA to estimate the proportion of variance explained by all SNPs [71]. The taxa were divided into two groups based on whether the taxa were present in ninety percent of participants. Our model was adjusted for age and sex. The power of GREML analysis was calculated with the GCTA power calculator [72].

#### Genome-wide association analysis of gut microbiome features

For each of the GNHS participants and the replication cohort, we clustered participants based on genus-level relative abundance, estimating the JSD distance and PAM clustering algorithm, and then we defined two enterotypes according to the Calinski-Harabasz index [34, 73]. We calculated the genetic principal components of ancestry from genome-wide genetic variants to estimate the population structure. PLINK 1.9 was used to perform a logistic regression model for enterotypes and taxa present in fewer than ninety percent, adjusted for age, sex, sequencing batch and the first five genetic principal components of ancestry.

For beta diversity, the analysis for the genome-wide host genetic variants with beta diversity was performed using MicrobiomeGWAS [35], adjusted for covariates including the first five genetic principal components of ancestry, age and sex.

Alpha diversity was calculated after randomly sampling 10,000 reads per sample. For the taxa present in no fewer than 90% of participants and alpha diversity, we used Z-score normalization to transform the distribution and carried out analysis based on a log-normal model. A mixed linear model-based association (MLMA) test in GCTA was used to assess the association, fitting the first five genetic principal components of ancestry, age, sex and sequencing batch as fixed effects and the effects of all the SNPs as random effects [74–76]. For other taxa present in fewer than 90% of participants, we transformed the absence/presence of the taxon into binary variables and used PLINK1.9 to perform a logistic model, adjusted for the first five genetic principal components of ancestry, age, sex and sequencing batch. For all the gut microbiome features, the significance threshold was defined as 5 × 10^−8^/*n* (*n* is the effective number of independent taxa in each taxonomic level) in the discovery stage. QUANTO software was used for power calculations (http://biostats.usc.edu/Quanto.html). We estimated genomic inflation factors with LDSC v1.0.1 at the local server [77].

#### Genetic correlation of gut microbiome and traits

We used GCTA to perform a bivariate GREML analysis to estimate the genetic correlation between the gut microbiome and traits in GNHS participants [74, 78]. The gut microbiome was divided into two groups according to the previous description. We used continuous variables for taxa present in no fewer than 90% of participants. For taxa present in fewer than 90% of participants, we used binary variables according to the absence/presence of taxa. This analysis included traits such as BMI, FBS, HbA1c, SBP, DBP, HDL-C, LDL-C, TC and TG. The power of bivariate GREML analysis was calculated with the GCTA power calculator [72].

#### Constructing polygenic scores for taxa and alpha diversity

We selected lead SNPs using PLINK v1.9 with the ‘—clump’ command to clump SNPs with a *p* value < 5 × 10^−5^ and *r*^2^ < 0.1 within 0.1 cM. We used beta coefficients as the weight to construct polygenic scores for taxa and alpha diversity. For alpha diversity and taxa present in no fewer than 90% of participants, we constructed weighted polygenic scores and performed the analysis on a general linear model with a negative binomial distribution to test for association between the polygenic scores and taxa, adjusted for the first five genetic principal components of ancestry, age, sex and sequencing batch. We used weighted polygenic scores and logistic regression to the absence/presence taxa, adjusted for the same covariates as in the above analysis. Taxa with significance (*p <* 0.05) in the replication cohort were included for further analysis.

### The effective number of independent taxa

As some taxa were correlated with each other, we used an eigendecomposition analysis to calculate the effective number of independent taxa for each taxonomic level [79, 80]. Matrix *M* is an *m* × *n* matrix, where *m* is the number of participants and *n* is the number of total taxa in the corresponding taxonomic level. Matrix *A* is the variance-covariance matrix of matrix *M*. *P* is the matrix of eigenvectors. diag{*λ*_1_, *λ*_2_, ⋯, *λ*_*n*_} is the diagonal matrix composed of the ordered eigenvalues, which can be calculated as

$$ \operatorname{diag}\left\{{\lambda}_1,{\lambda}_2,\cdots, {\lambda}_n\right\}={P}^{-1} AP $$

The effective number of independent taxa can be calculated as

$$ \frac{{\left({\sum}_{i=1}^n{\lambda}_i\right)}^2}{\sum_{i=1}^n{\lambda}_i^2} $$

### Bidirectional MR analysis

In the analysis of the potential causal effect of gut microbiome features on diseases, we used independent genetic variants (selected as part of the polygenic score analysis) as the instrumental variables. For each trait, we excluded instrumental variables that showed a significant association with the trait (*p <* 0.05/*n*, where *n* is number of independent genetic variants). In the analysis of the potential causal effect of diseases on gut microbiome features, we selected genetic variants that were replicated in East Asian populations as instrumental variables. As all instrumental variables were from East Asian populations, we chose independent genetic variants (*r*^2^ < 0.1) based on the GNHS cohort. We identified the best proxy (*r*^2^ > 0.9) based on the GNHS cohort or discarded the variant if no proxy was available. We used the inverse variance weighted (IVW) method to estimate the effect size. To confirm the robustness of the results, we performed three other MR methods, including weighted median, MR-Egger and MR-PRESSO [81–83]. To assess the presence of horizontal pleiotropy, we performed the MR-PRESSO global test and MR-Egger regression. The magnitude of the effect of the gut microbiome on traits was dependent on the units of traits (Supplementary Table S1). The results of the effects of complex human diseases on the absence/presence of specific gut microbes are presented as the risk of the presence (vs. absence) of the microbe per the log odds difference of the disease. The results of the effects of diseases on other gut microbes were presented as changes in the abundance of taxa (1-SD of log transformed) per the log odds difference of the respective disease.

The statistical significance of the effects of the gut microbiome on traits and diseases was defined as *p <* 0.0008 (0.05/62). In addition, the statistical significance of the effects of diseases on gut microbiome features was defined as *p <* 0.05/*n* (where *n* is the effective number of independent taxa on the corresponding taxonomic level). The results that could not pass Bonferroni adjustment but *p <* 0.05 in all four MR methods were considered potential causal relationships. We performed MR analyses with R v3.5.3.

### Pathway analysis

We used OTUs by QIIME and annotated the variation of functional genes with Phylogenetic Investigation of Communities by Reconstruction of Unobserved States (PICRUSt) [39]. The pathways and diseases were annotated using KEGG [84–86]. We used Spearman’s rank-order correlation to investigate the association of the predicted pathway or disease abundance with AF-associated taxa and genus-level taxa. In the heatmap, diseases were clustered with the ‘hcluster’ function in R. To test whether the non-normalized pathway or disease abundance were associated with each other, we used SPIEC-EASI to test the interaction relationship and then used Cytoscape v3.7.2 to visualize the interaction network [87, 88].

### Construction of the microbiome risk score

The microbiome risk score was constructed to validate the accuracy of the association between the predicted disease-related gut microbiome features and the corresponding disease. As we have a large sample size for T2DM cases (*n* = 217 cases) in our cohort, we constructed a microbiome risk score of T2DM as an example. We used Spearman’s rank-order correlation to select taxa with an absolute value of correlation coefficient higher than 0.2. The score for each taxon abundance in the < 5% quantile in our study was defined as 0. For those above 5%, the score for each taxon showing an inverse association with T2DM was defined as − 1; the score for each taxon showing a positive association with T2DM was defined as 1. We then summed values from all taxa. We selected a logistic regression model to estimate the association of the MRS with T2DM risk and a linear model to estimate the association of the MRS with the continuous variables, adjusted for age, sex, dietary energy intake, alcohol intake and BMI at the time of sample collection.

### Clustering diseases

The clustering analysis was carried out with ‘cluster’ and ‘factoextra’ for plot in R. We performed the PAM algorithm based on the predicted abundance of diseases or the average relative abundance after Z-score normalization [89]. The PAM algorithm searches *k* medoids among the observations and then finds the nearest medoids to minimize the dissimilarity among clusters [90]. Given a set of objects *x* = (*x*_1_, *x*_2_, …, *x*_*n*_), the dissimilarity between objects *x*_*i*_ and *x*_*j*_ is denoted by d(*i*, *j*). The assignment of object *i* to the representative object *j* is denoted by *z*_*ij*_. *z*_*ij*_ is a binary variable and is 1 if object *i* belongs to the cluster of the representative object *j*. The function to minimize the model is given by

$$ \sum \limits_{i=1}^n\sum \limits_{j=1}^nd\left(i,j\right){z}_{ij} $$

To identify the optimal cluster number, we used the ‘pamk’ function in R to determine the optimum average silhouette width. For each object *i*, we defined *N*_*i*_ as the average dissimilarity between object *i* and all other objects within its cluster. For the remaining clusters, b(*i*, *w*) represents the average dissimilarity between *i* and all objects in cluster *w*. The minimum dissimilarity *M*_*i*_ can be calculated by

$$ {M}_i=\mathit{\min}\forall w\left(b\left(i,w\right)\right). $$

The silhouette width for object i can be calculated by

$$ {sw}_i=\frac{M_{i\kern0.75em }-{N}_i}{\mathit{\max}\left({M}_{i\kern0.75em },{N}_i\right)} $$

Then, we calculated the average silhouette width for each object. The cluster number is determined by the number at which the average silhouette width is maximum. We estimated the Jaccard similarity coefficient to quantify the cluster difference between groups. The Jaccard similarity coefficient is positively associated with the similarity of clusters. Given objects *i* and *j*, as well as groups A and B, there are four situations, as follows:

1. S1: in both groups A and B, objects *i* and *j* belong to the same cluster
2. S2: in group A, objects *i* and *j* belong to the same cluster; in group B, they belong to different clusters
3. S3: in group A, objects *i* and *j* belong to different clusters; in group A, they belong to the same cluster
4. S4: in both groups A and B, objects *i* and *j* belong to different clusters

The letters a, b, c and d represent the numbers of S1, S2, S3 and S4, respectively. The Jaccard similarity coefficient can be calculated by the following formula:

$$ J=\frac{a}{a+b+c} $$

## Supplementary information

**Additional file 1: Table S1.** Transformation of traits in BioBank Japan and taxa in GNHS. **Table S2.** Required effect size (beta) to reach 80% of power in GNHS cohort. **Table S3** Heritability of taxa, enterotype and alpha diversity. **Table S4.** Significant genetic correlations of gut microbiome and metabolic traits. **Table S5.** Significant associations of all taxa with SNPs identified in the discovery stage before adjustment (*p*<5e-8). **Table S6.** Replication of genetic variants associated with taxa. **Table S7.** Replication of genetic variants associated with beta diversity. **Table S8.** Replication of genetic variants associated with alpha diversity. **Table S9.** Lead SNPs used to construct polygenic scores. **Table S10.** MR analysis of gut microbiota on traits and diseases. **Table S11.** MR analysis of diseases on gut microbiota features. **Table S12.** Spearman’s correlation of certain taxa and complex diseases. **Table S13.** Spearman’s correlation of gut microbiota on genus level and characteristics.

**Additional file 2: Figure S1.** Genome-wide analysis results of taxa. **Figure S2.** Spearman’s correlation of the relative abundance of AF-associated taxa with the relative level of diseases predicted by PICRUSt.

## Data Availability

The raw data for 16S rRNA gene sequences are available in the CNSA (https://db.cngb.org/cnsa/) of CNGBdb at accession number CNP0000829. Original R scripts are available in GitHub (https://github.com/hsufengzhe/microbiome/tree/master). Requests for the metadata from this study can be submitted via email to zhengjusheng@westlake.edu.cn. A proposal is also required for approval.
