# Supplementary material for: The interplay between host genetics and the gut microbiome reveals common and distinct microbiome features for complex human diseases
Source: Microbiome. 2020 Oct 8;8:145. doi: 10.1186/s40168-020-00923-9 (PMC7545574; doi:10.1186/s40168-020-00923-9)

Supplementary Figure 1 Genome-wide analysis results of taxa. The red line in Manhattan plot indicates the threshold of 5×10-8 and the blue line indicates the threshold of 5×10-5. Quantile-quantile plot (QQ-plot) shows observed –log10 p-values with the expected –log10 p-values. Blue bots show all SNPs and the red line indicates null hypothesis of no association. (A). Manhattan plot of all SNPs from genome-wide analysis of *Saccharibacteria*. (B). QQ-plot of all SNPs genome-wide analysis of *Saccharibacteria*. (C). Manhattan plot of all SNPs from genome-wide analysis of *Clostridiaceae*. (D). QQ-plot of all SNPs genome-wide analysis of *Clostridiaceae*. (E). Manhattan plot of all SNPs from genome-wide analysis of *Comamonadaceae*. (F). QQ-plot of all SNPs genome-wide analysis of *Comamonadaceae*. (G). Manhattan plot of all SNPs from genome-wide analysis of *Klebsiella*. (H). QQ-plot of all SNPs genome-wide analysis of *Klebsiella*. (I). Manhattan plot of all SNPs from genome-wide analysis of *Desulfovibrio d168*. (J). QQ-plot of all SNPs genome-wide analysis of *Desulfovibrio d168*.

Supplementary Figure 2 Spearman's correlation of the relative abundance of AF-associated taxa with the relative level of diseases predicted by PICRUSt. The grey components show no significance of correlation with Bonferroni correction (p>0.05/ (5.6*22), p>0.0004).

Supplementary Figure 1 Genome-wide analysis results of taxa. The red line in Manhattan plot indicates the threshold of 5×10-8 and the blue line indicates the threshold of 5×10-5. Quantile-quantile plot (QQ-plot) shows observed –log10 p-values with the expected –log10 p-values. Blue bots show all SNPs and the red line indicates null hypothesis of no association. (A). Manhattan plot of all SNPs from genome-wide analysis of *Saccharibacteria*. (B). QQ-plot of all SNPs genome-wide analysis of *Saccharibacteria*. (C). Manhattan plot of all SNPs from genome-wide analysis of *Clostridiaceae*. (D). QQ-plot of all SNPs genome-wide analysis of *Clostridiaceae*. (E). Manhattan plot of all SNPs from genome-wide analysis of *Comamonadaceae*. (F). QQ-plot of all SNPs genome-wide analysis of *Comamonadaceae*. (G). Manhattan plot of all SNPs from genome-wide analysis of *Klebsiella*. (H). QQ-plot of all SNPs genome-wide analysis of *Klebsiella*. (I). Manhattan plot of all SNPs from genome-wide analysis of *Desulfovibrio d168*. (J). QQ-plot of all SNPs genome-wide analysis of *Desulfovibrio d168*.

A

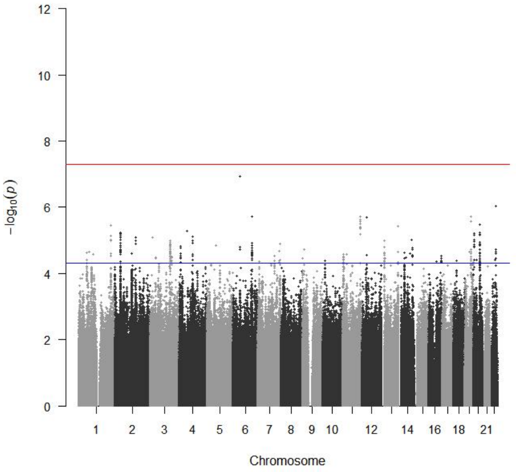

B

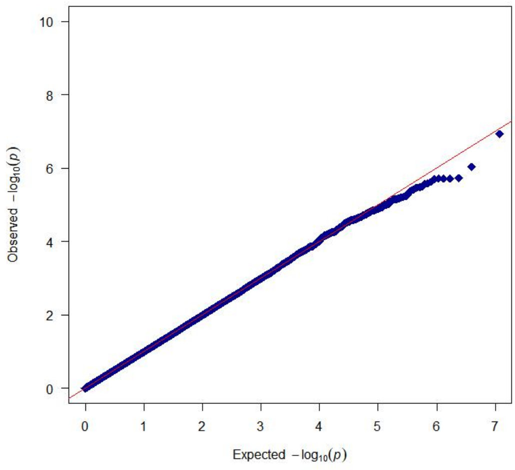

C

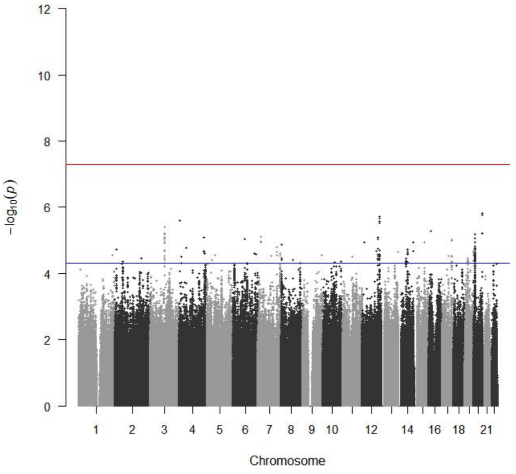

D

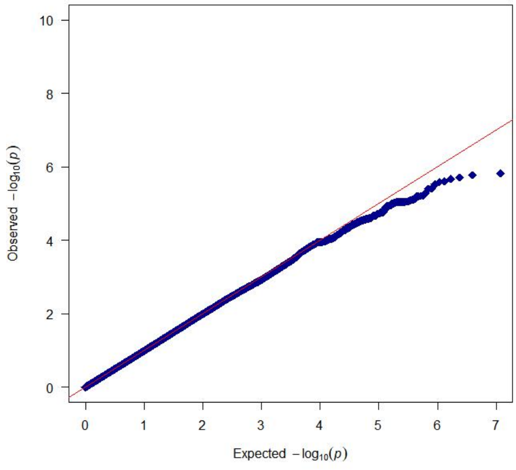

E

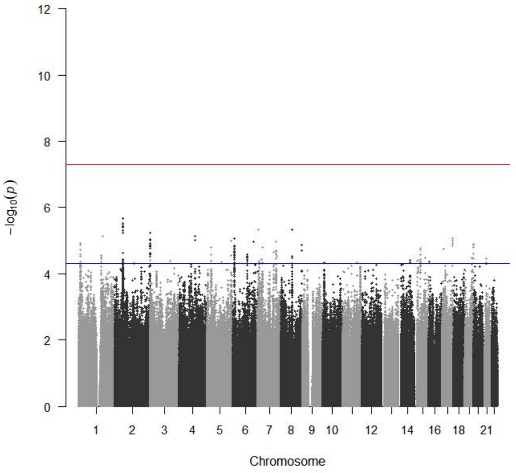

$-\log_{10}(p)$
Chromosome
1 2 3 4 5 6 7 8 9 10 12 14 16 18 21

F

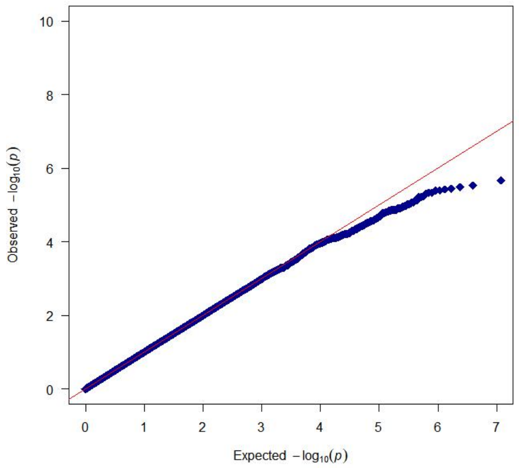

Observed $-\log_{10}(p)$
Expected $-\log_{10}(p)$

G

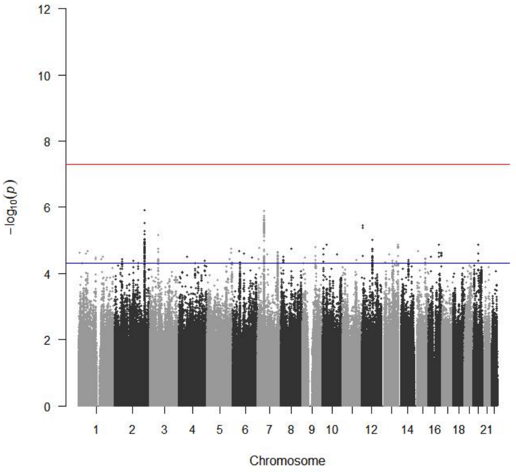

$-\log_{10}(p)$
Chromosome
1 2 3 4 5 6 7 8 9 10 12 14 16 18 21

H

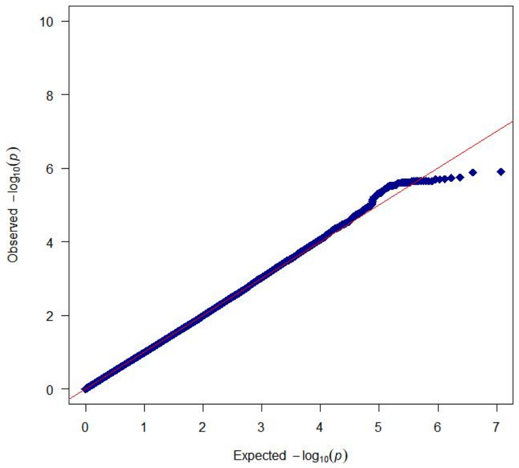

Observed $-\log_{10}(p)$
Expected $-\log_{10}(p)$

I

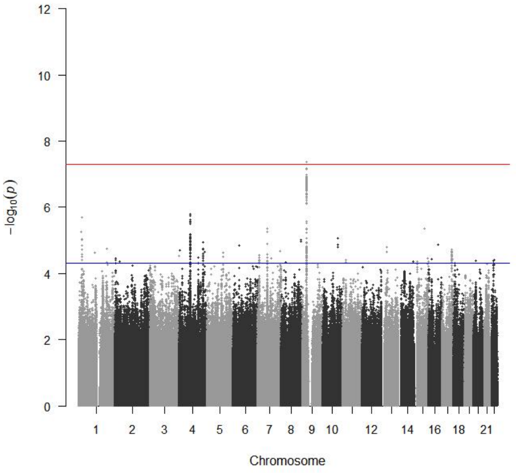

$-\log_{10}(p)$
Chromosome
1 2 3 4 5 6 7 8 9 10 12 14 16 18 21

J

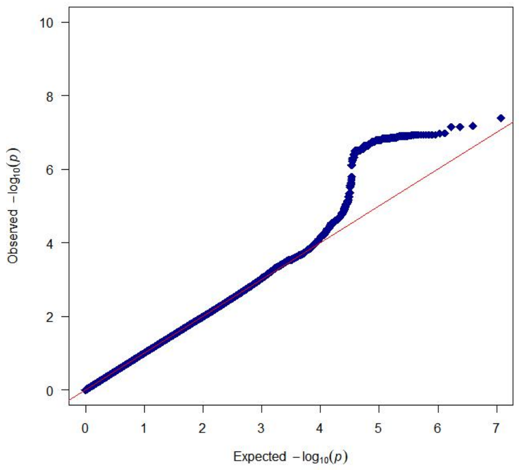

Observed $-\log_{10}(p)$
Expected $-\log_{10}(p)$

Supplementary Figure 2 Spearman's correlation of the relative abundance of AF-associated taxa with the relative level of diseases predicted by PICRUSt. The grey components show no significance of correlation with Bonferroni correction (p>0.05/ (5.6*22), p>0.0004).

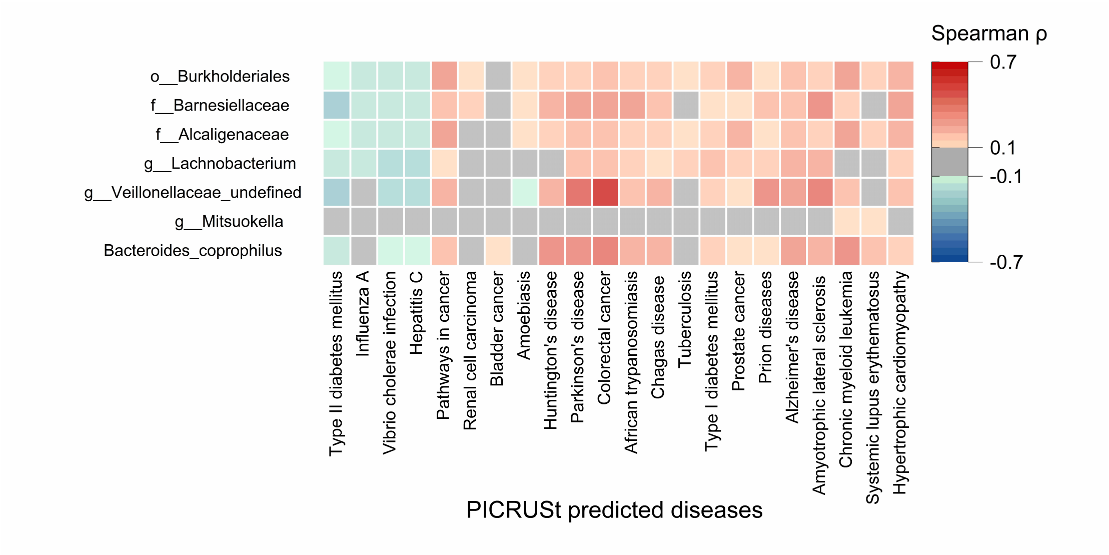

Supplement: Supplementary file 3 — Additional file 2: Figure S1. Genome-wide analysis results of taxa. Figure S2. Spearman’s correlation of the relative abundance of AF-associated taxa with the relative level of diseases predicted by PICRUSt. [file 40168_2020_923_MOESM2_ESM.pdf]
